# Melanoma Chemotherapy Leads to the Selection of ABCB5-Expressing Cells

**DOI:** 10.1371/journal.pone.0036762

**Published:** 2012-05-24

**Authors:** Marine Chartrain, Joëlle Riond, Aline Stennevin, Isabelle Vandenberghe, Bruno Gomes, Laurence Lamant, Nicolas Meyer, Jean Edouard Gairin, Nicolas Guilbaud, Jean Philippe Annereau

**Affiliations:** 1 UMR 2587, CNRS-Pierre Fabre, Institut des Sciences et Technologies du Médicament de Toulouse (ISTMT), Toulouse, France; 2 Centre de Recherche en Oncologie Expérimentale, Centre de Recherche et Développement Pierre Fabre - Toulouse Langlade, Toulouse, France; 3 USR 3388, CNRS-Pierre Fabre, Centre de Recherche et Développement Pierre Fabre - Toulouse Langlade, Toulouse, France; 4 INSERM U563, CHU Purpan, Toulouse, France; 5 Hôpital Larrey, Toulouse, France; 6 UMR152 IRD-UPS “PHARMA-DEV”, Faculté de Pharmacie - Université Toulouse III - Paul Sabatier, Toulouse, France; Wistar Institute Program, United States of America

## Abstract

Metastatic melanoma is the most aggressive skin cancer. Recently, phenotypically distinct subpopulations of tumor cells were identified. Among them, ABCB5-expressing cells were proposed to display an enhanced tumorigenicity with stem cell-like properties. In addition, ABCB5^+^ cells are thought to participate to chemoresistance through a potential efflux function of ABCB5. Nevertheless, the fate of these cells upon drugs that are used in melanoma chemotherapy remains to be clarified. Here we explored the effect of anti-melanoma treatments on the ABCB5-expressing cells. Using a melanoma xenograft model (WM266-4), we observed *in vivo* that ABCB5-expressing cells are enriched after a temozolomide treatment that induces a significant tumor regression. These results were further confirmed in a preliminary study conducted on clinical samples from patients that received dacarbazine. *In vitro*, we showed that ABCB5-expressing cells selectively survive when exposed to dacarbazine, the reference treatment of metastatic melanoma, but also to vemurafenib, a new inhibitor of the mutated kinase V600E BRAF and other various chemotherapeutic drugs. Our results show that anti-melanoma chemotherapy might participate to the chemoresistance acquisition by selecting tumor cell subpopulations expressing ABCB5. This is of particular importance in understanding the relapses observed after anti-melanoma treatments and reinforces the interest of ABCB5 and ABCB5-expressing cells as potential therapeutic targets in melanoma.

## Introduction

Melanoma is one of the most aggressive form of skin cancer and its incidence is increasing worldwide, especially where fair-skinned people receive excessive sun exposure [1]–[3]. Primary tumors without any evidence of metastases are mostly treated by surgery. However, metastatic melanoma is highly resistant to conventional radio and chemotherapies and remains a disease of poor prognosis, with median survival times comprised between 7 and 9 months. A number of chemotherapeutic agents (such as dacarbazine, temozolomide or fotemustine) alone or in combination have a limited activity with relatively low response rates (<25% for any single agent) [4], [5] and so far only a small impact on overall survival. Even if some promising targeted therapies are currently developed with new BRAF kinase inhibitors such as vemurafenib [6], melanomas invariably become resistant to these agents [7]. Thus, chemoresistance remains a serious concern for melanoma therapy.

Besides the mechanisms of resistance to chemotherapy that are shared by various cancers, melanomas display specific features. Melanoma cells are equipped with melanogenesis-related vesicles, the melanosomes, that have been shown to be involved in drug trapping and export [8]. Secondly, these cells express ABC transporters which have been associated with multidrug resistance by lowering the intracellular accumulation of cytotoxic drugs [9]. Of particular interest is ABCB5 which shares 73% of sequence homology with ABCB1 (P-gp, MDR1) [10], [11]. Firstly detected in tissues derived from the neuroectodermal lineage including melanocyte progenitors [10], melanoma cell lines and patient specimens [11]–[14], ABCB5 expression was also found in other tissues [15]–[17] but is restricted to a subpopulation of cells. In melanoma, ABCB5-expressing cells are endowed with self-renewal, differentiation and tumorigenicity abilities [18], [19]. Their abundance in clinical melanoma specimens correlates positively with the neoplasic progression suggesting that ABCB5 expression is associated with tumor aggressiveness. Moreover, the growth of melanoma xenografts in mice was delayed when the animals were treated with a monoclonal anti-ABCB5 antibody [18].

As a member of the ABC transporter family, ABCB5 is thought to play a role in drug efflux. This was supported by experiments measuring the intracellular accumulation of Rhodamine 123 [10] or doxorubicin in melanoma [14] and hepatocarcinoma cells [15], [16]. Nevertheless, the level of resistance of melanoma ABCB5-expressing cells to relevant chemotherapeutic drugs remains unknown. Here, we demonstrated that ABCB5^+^ cells display a survival advantage over ABCB5^−^ cells upon anti-melanoma treatment. We show that: 1) ABCB5-expressing cells selectively survive over ABCB5^−^ cells after a temozolomide treatment inducing a significant tumor regression in the WM-266-4 xenograft model 2) ABCB5-expressing cells are more abundant in melanomas from patients treated with dacarbazine 3) *in vitro*, dacarbazine but also vemurafenib and other drugs induce an increase in the ABCB5-expressing cell population at doses that are cytotoxic for the bulk cells.

## Results

### ABCB5 Expression is Restricted to a Subpopulation of Melanoma Cells

**Figure 1:**
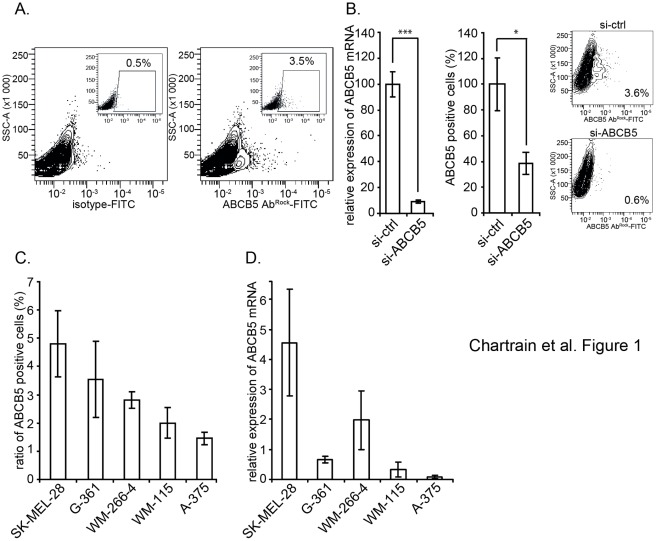
ABCB5 is expressed on the surface of a subpopulation of melanoma cells. WM-266-4 cells were surface-labelled with the ABCB5-Ab^Rock^ antibody and analyzed by flow cytometry. ABCB5^+^ cells (right contour plot) were gated on viable cells (DAPI-negative) according to the isotype control (left contour plot). Inserts (dot plots) display the gating of the positive cells (**A**). WM-266-4 cells were treated with a siRNA designed to target ABCB5 (si-ABCB5). After 72 h, the cells were analyzed for their ABCB5 mRNA content and ABCB5 surface expression. The left and right histograms show respectively the relative expression of ABCB5 mRNA normalized to the ABCB5 mRNA in cells treated with a control siRNA (si-ctrl), and the percentage of ABCB5^+^ cells among total cells (n = 3). The corresponding contour plots are shown (**B**). Different melanoma cell lines were analyzed for their ABCB5 surface expression (**C**) or their ABCB5 mRNA content (**D**) (n = 3).

To assay the variation of the expression of ABCB5, we first devised the methodology to detect the presence of ABCB5-expressing cells in a panel of melanoma cell lines using a rabbit polyclonal antibody raised against a peptide derived from the ABCB5 protein sequence (ABCB5-Ab^Rock^). In the WM-266-4 cell line, this antibody consistently labels a subpopulation of living cells that can be distinguished among the bulk of cells by flow cytometry (figure 1A). The proportion of positive cells is low but significant and reproducible within the repeated experiments. The specificity of the labelling was assessed by depletion experiments using RNAi interference. The siRNA-mediated down-regulation of the ABCB5 mRNA expression, as measured by Q-PCR, was associated with a strong reduction of the ABCB5^+^ population to 37% of the control (figure 1B). In addition, we confirmed that ABCB5-expressing cells detected with the ABCB5-Ab^Rock^ antibody are also labelled by the monoclonal 3C2-1D12 one (ABCB5-Ab^AbD^) described earlier [10], [14] (data not shown).

The presence of such an ABCB5^+^ subpopulation is not restricted to the WM-266-4 cell line model and was detected in most of the tested melanoma cell lines, with a proportion ranging from 1 to 5% of the total cells (figure 1C). Interestingly, the amounts of the ABCB5 mRNA quantified by Q-PCR and the percentages of the ABCB5-expressing cells appear to be correlated for four of the five tested cell lines (figure 1C and D), with the exception of G361 cells in which an ABCB5^+^ subpopulation was detected despite a relatively low abundance of mRNA.

Of note, the WM-115 cell line derived from a primary melanoma tumor expresses six times less ABCB5 mRNA than the WM-266-4 cell line which originates from a metastasis of the same patient.

### ABCB5-expressing Cells are Enriched in the Residual Tumors after an Anti-melanoma Treatment *in vivo*

**Figure 2:**
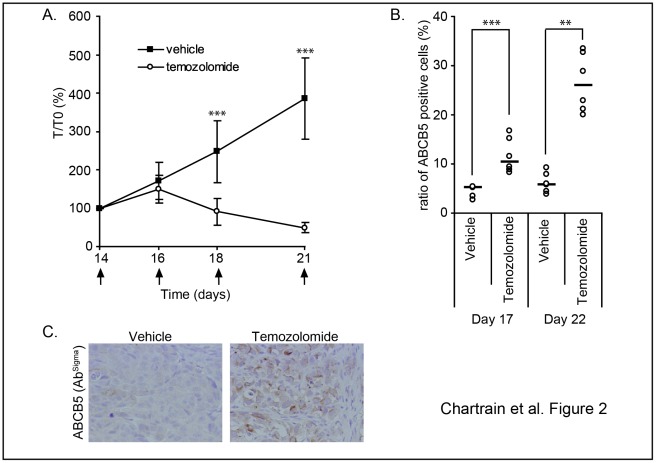
ABCB5-expressing cells are enriched in the residual tumors after an anti-melanoma treatment *in vivo.* WM-266-4 cells (5×10^6^ cells) were injected subcutaneously in Swiss nude mice. Fourteen days later, mice were treated by repeated i.p. injections of either temozolomide (80 mg/kg) or vehicle following the schedule indicated by the black arrows. The tumoral volumes were monitored and the means of measured volumes respectively for temozolomide-treated and vehicle treated tumors are as follows: 180 mm^3^ and 175 mm^3^ at day 14; 264 mm^3^ and 295 mm^3^ at day 16; 178 mm^3^ and 444 mm^3^ at day 18; 84 mm^3^ and 699 mm^3^ at day 21. (**A**). 24 h after the injections at days 16 and 21 (d17 and d22), tumors were recovered, dissociated and the cell suspensions were searched for the presence of human ABCB5^+^ cells by flow cytometry (**B**) (medians are represented as black lines). Tumors recovered at day 17 were analyzed by immunohistochemistry for their ABCB5 expression (**C**).

We investigated the effect of an anti-melanoma chemotherapeutic treatment on the ABCB5-expressing cell subpopulation *in vivo*. This was assayed using WM-266-4 tumor cells subcutaneously xenografted in nude mice. Temozolomide was selected as the anti-melanoma substance. Mice bearing 14-days tumors were treated at days 14, 16, 18, 21 with the drug doses known to induce a significant anti-tumor effect. Tumor growth was followed over a 21 days period (figure 2A) and tumor specimens were harvested and analyzed for their content in ABCB5-expressing cells at days 17 and 22. As shown on figure 2B, an increase in the ABCB5^+^ cells ratio is detected 24 h after the second injection of temozolomide, concomitantly with an efficient tumor growth inhibition. This ratio increases again at day 22 after two more drug administrations when a severe mass reduction is observed. Immunohistochemical analysis of mice tumors with a different antibody confirms an increased expression of ABCB5 in tumors from the treated mice (figure 2C).

### ABCB5 Expression is Enhanced after Treatment in Clinical Samples

**Figure 3:**
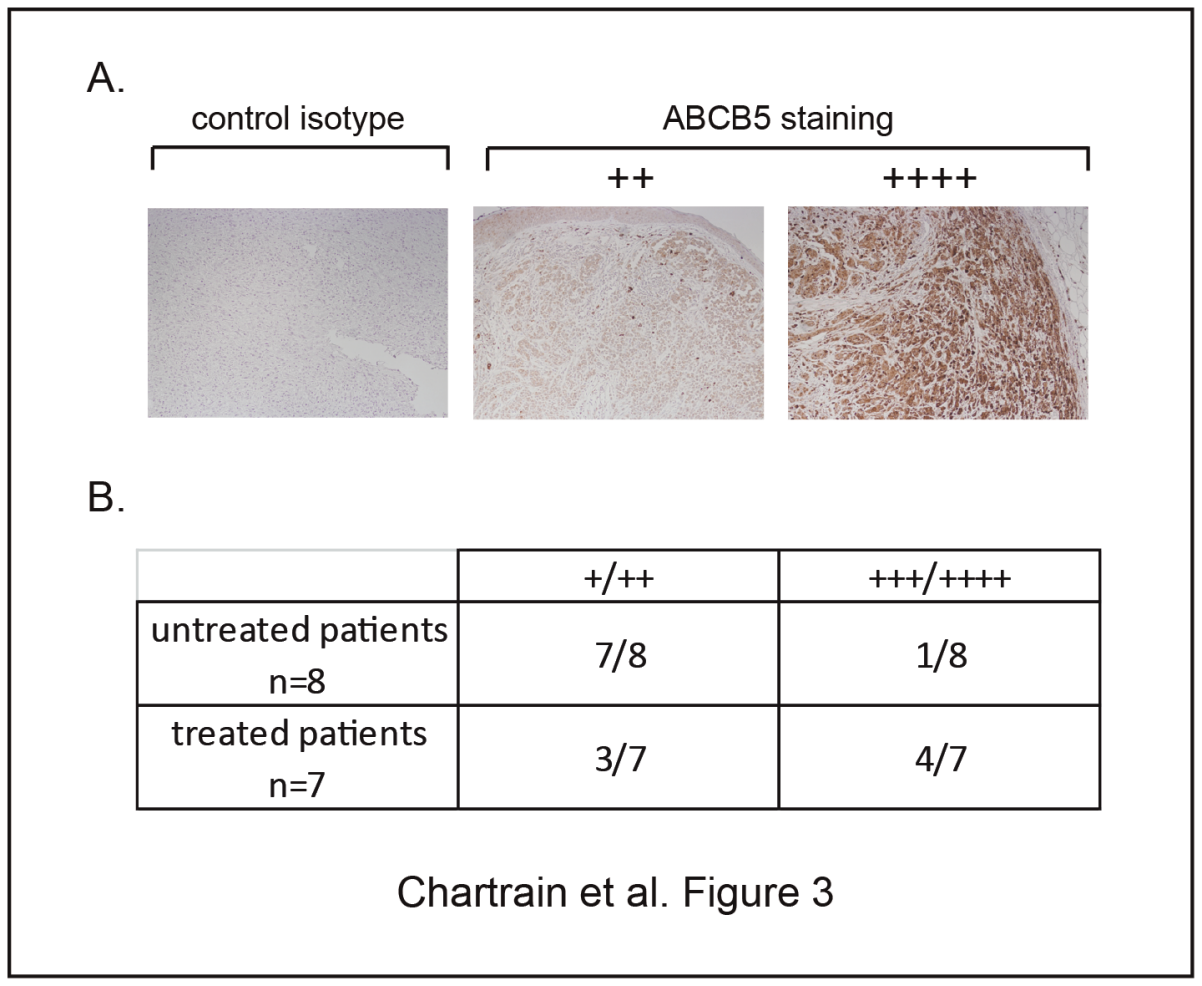
ABCB5 expression is increased in melanoma tumors obtained from treated patients. Skin metastases specimens from respectively 8 untreated and 7 treated patients were analyzed by immunohistochemistry for their ABCB5 protein expression. The ABCB5 staining intensity was ranked in four arbitrary classes according to the intensity and the extent of the labelling. Representative staining of two levels of intensity (left panel: isotypic control) (A). Repartition of the specimens in the different classes (B). The two groups of specimens (untreated versus treated) have been compared with the non parametric Kruskall Wallis test (p<0.30).

We analyzed the expression of ABCB5 in human melanoma metastatic samples obtained from unmatched patients, before and after treatment. The figure 3 shows that 7 of the 8 samples from untreated patients display low-staining intensity (on a level-scale based on the extent and intensity of the staining on the whole tumor section). By contrast, 4 of the 7 tumors from treated patients display a higher level of ABCB5 staining. While this difference is not statistically significant due to the low number of examined samples, it nevertheless suggests a higher expression of ABCB5 in tumors from patients having received treatment.

### ABCB5-expressing Cells Enrichment upon Cytotoxic Treatment is Due to their Selective Survival

**Figure 4:**
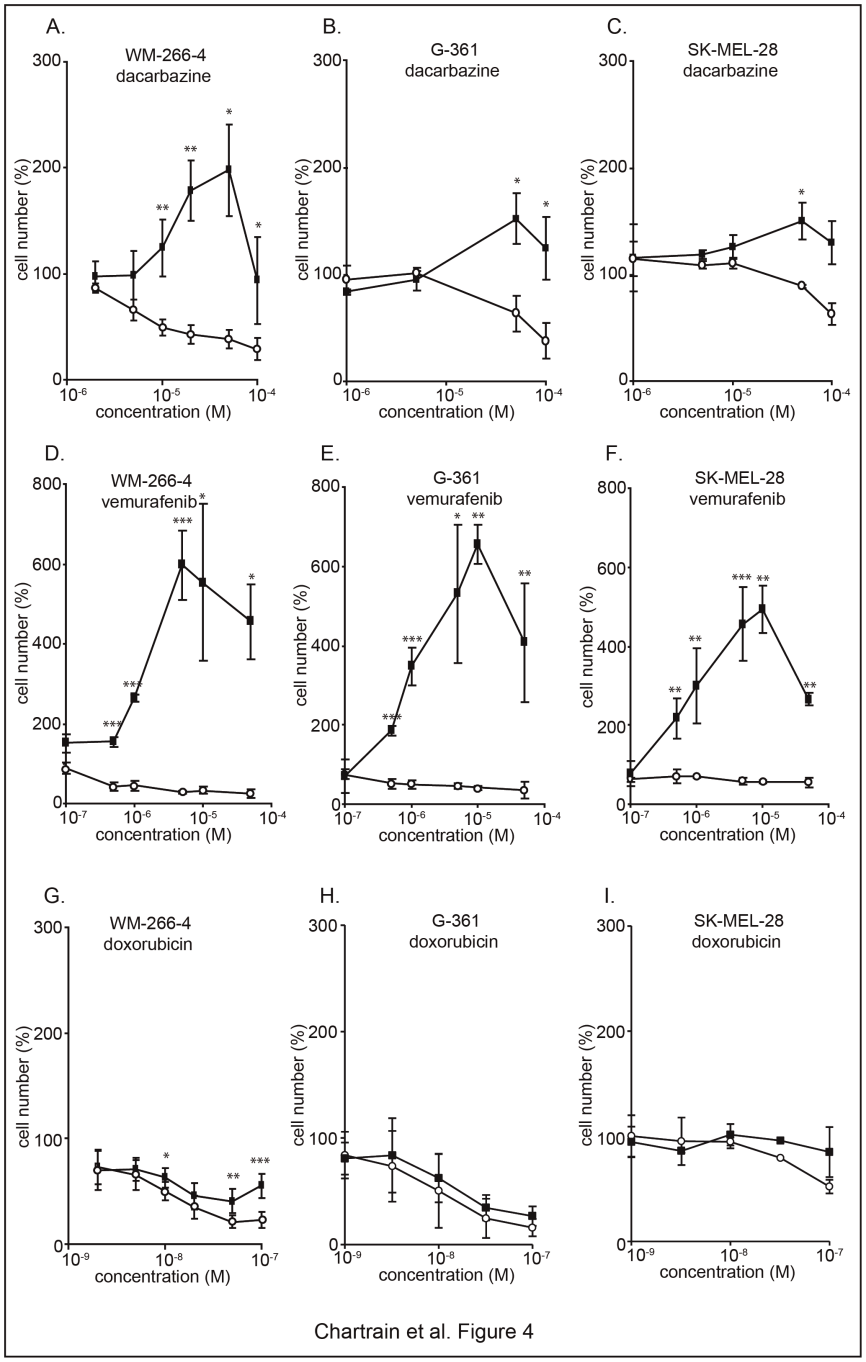
ABCB5-expressing cells survive upon dacarbazine treatment. WM-266-4 (**A,D,G**), G-361 (**B,E,H**) and SK-MEL-28 cells (**C,F,I**) were treated at the indicated concentrations of dacarbazine (**A–C**), vemurafenib (**D–F**) and doxorubicin (**G–I**). After 72 h, the total viable cells were numbered using an automated cell viability analyzer. The percentages of viable ABCB5-expressing cells (among viable cells gated on DAPI-negative cells) were analyzed by flow cytometry. The numbers of total cells (white symbols) are reported as percentages of the number of cells in the untreated control sample. The numbers of viable ABCB5^+^ cells (black symbols) were calculated from the total cell numbers and ABCB5^+^ cells ratio, and reported as percentages of the viable ABCB5^+^ cells number in the control sample. ABCB5^+^ cells represent respectively 3%, 3.5% and 5% of the total cells in the WM-266-4, G-361 and SK-MEL-28 cell lines.

We aimed at understanding the origin of the ABCB5-expressing cells enrichment we observed *in vivo*. WM-266-4 cells were treated *in vitro* with dacarbazine which is structurally related to temozolomide and is active *in vitro*. Total and ABCB5^+^ cells numbers were monitored simultaneously after a 72 h treatment, using a range of cytotoxic drug concentrations. While the number of the bulk cells decreases with increasing concentrations of drug, the number of ABCB5^+^ cells remains above their basal number in untreated sample (figure 4A). The survival advantage of ABCB5^+^ cells was also found in G-361 and SK-MEL-28 cell lines (figure 4B and 4C) and was confirmed with a different antibody (data not shown). In addition to the survival, an increase in ABCB5^+^ cell numbers were detected to a certain extent in the different cell lines analyzed. This was particularly clear in the WM-266-4 cells in which ABCB5^+^ cells are increased by a two-fold factor (figure 4A). The dose-response profile is biphasic, with a maximum effect at a concentration that induces 50–75% of cytotoxicity on the bulk cells. At higher concentrations, the ABCB5^+^ cells numbers decrease and reach the basal control value (100%). The same response profiles were obtained when melanoma cells were treated with vemurafenib (figure 4D–4F). Moreover, the numbers of ABCB5^+^ cells detected upon vemurafenib are significantly higher than with dacarbazine. By contrast, under doxorubicin exposure, the numbers of ABCB5^+^ cells decrease with increasing concentrations suggesting that they are sensitive to the cytotoxic treatment. Nevertheless, they display a slight survival advantage over the bulk cells (figure 4G–4I).

### Dacarbazine Treatment Stimulates the Neo-synthesis of the ABCB5 Protein and its Exposure at the Cell Surface

**Figure 5:**
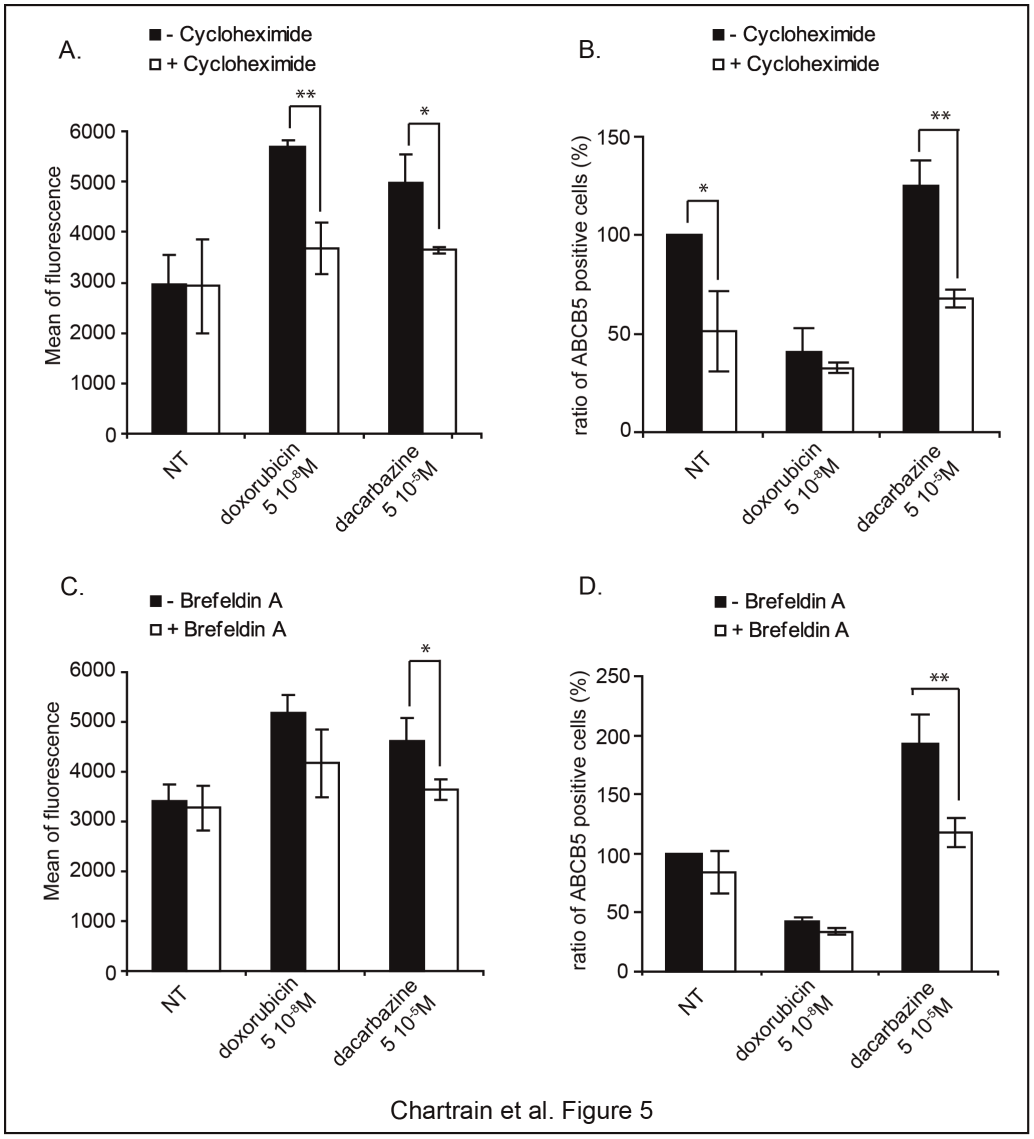
Enrichment in ABCB5-expressing cells is associated with protein neo-synthesis and ABCB5 relocation at the cell-surface. WM-266-4 cells were treated with dacarbazine, doxorubicin or vehicle (NT) for 72 h. Cycloheximide (**A–B**) or brefeldin A (**C–D**) were added respectively 24 h and 4 h before the treatment end-point. Cells were labelled for ABCB5 and analyzed by flow cytometry. The means of fluorescence intensity of the ABCB5^+^ cells from three independent experiments were reported in **A** and **C**. Fluorescence intensity histograms of representative experiments are shown in the figure S1. The number of ABCB5^+^ cells was reported in **B** and **D** as a percentage of the ABCB5^+^ cell number in the vehicle-treated sample.

The results shown above suggest that, in addition to being less cytotoxic for ABCB5^+^ cells than for the bulk cells, dacarbazine has other effects that participate to increase the ABCB5^+^ cells number detected after treatment. In order to determine a possible role of protein neo-synthesis and vesicular trafficking, we studied the effect of cycloheximide and brefeldin A on ABCB5 expression. We measured both the level of ABCB5 at the cell surface via the fluorescence intensity and the number of cells expressing a detectable level of ABCB5 via the percentage of ABCB5^+^ cells (figure 5 and figure S1). Cycloheximide does not affect the basal level of ABCB5 at the surface of untreated cells, but reverses its increase when cells are treated with either dacarbazine or doxorubicin (figure 5A). Concomitantly, it reduces the percentage of ABCB5^+^ cells in untreated cells or in dacarbazine-treated cells. Cycloheximide has no significant impact on the number of ABCB5^+^ cells after doxorubicin treatment (figure 5B).

Brefeldin A has no effect on either the basal expression of ABCB5 or the basal number of detected ABCB5^+^ cells in untreated cells. But it reduces both parameters in dacarbazine-treated cells suggesting that trafficking participates to increase the ABCB5 expression at the cell surface. No significant effect was seen with doxorubicin.

### Selective ABCB5-expressing Cells Survival upon Dacarbazine Occurs with Other Clinically Used Drugs and is Unrelated to ABCB1 Expression

**Figure 6:**
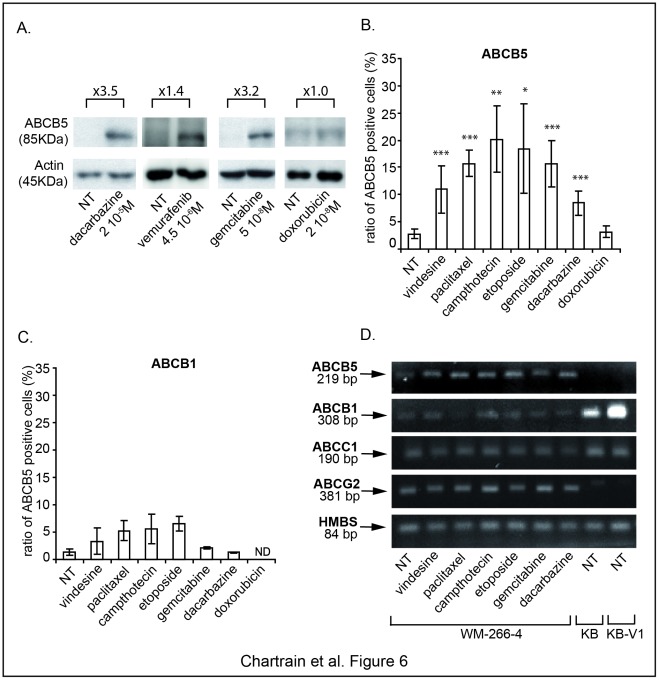
Quantification of ABCB5- and ABCB1-expressing cells after cytotoxic treatments. WM-266-4 cells were treated for 72 h with the indicated concentrations of doxorubicin, dacarbazine, vemurafenib, gemcitabine or with vehicle (NT) and ABCB5 expression was analyzed by Western blot. Band intensities were quantified and variations are indicated as fold increases in treated versus untreated samples (**A**). WM-266-4 cells were treated with various drugs at their EC50 for 72 h. The percentages of positive cells among surviving cells were measured by cell surface labelling and flow cytometry analysis for ABCB5 (**B**) or ABCB1 (**C**). The relative mRNA expression of ABCB5, ABCB1, ABCC1, ABCG2 and HMBS as the house-keeping gene was measured by Q-PCR (see also table S1) and the amplified products were run on agarose gel after 29 cycles except for ABCB1 (32 cycles) (**D**).

The ABCB5 protein is barely detectable in untreated WM-266-4 cells by Western blot analysis (figure 6A). By contrast, treatment with dacarbazine, vemurafenib or gemcitabine (which also leads to enrichment of ABCB5-expressing cells) induces an increased signal corresponding to a protein at the expected molecular weight of 85 kDa. Concordantly with the lack of increase of ABCB5^+^ cells under doxorubicin exposure, no enrichment of the ABCB5 protein was seen in doxorubicin-treated samples.

We analyzed the effect of different other drugs on ABCB5-expressing cells. The percentages of ABCB5^+^ cells were monitored by flow cytometry after 72 h-treatments at their respective EC50 (figure 6B). All the tested drugs except doxorubicin increase by a factor 3 to 9 the percentage of ABCB5^+^ cells among the surviving cells.

Because the expression of different ABC transporters has been observed in melanoma, we evaluated their potential implication in the ABCB5^+^ cells survival upon dacarbazine as well as other chemotherapeutic agents. As shown by flow cytometry experiments (figure 6C), ABCB1 is expressed on a very rare subpopulation in untreated cells. By contrast with the ABCB5-expressing cells, the ABCB1^+^ subpopulation is not enriched after a dacarbazine (or gemcitabine) treatment. Non significant increases in the percentages of ABCB1^+^ cells were found after treatment with vindesine, paclitaxel, camptothecin and etoposide. Q-PCR experiments confirmed the low level of ABCB1 expression in the WM-266-4 cells compared to the ABCB1^+^ cells KB and KB-V1 (figure 6D and table S1). Some variations were detected in the ABCB1 expression after drug treatment, but they are not statistically significant.

The expression of ABCC1 and ABCG2 was also measured by Q-PCR. While these transporters are more abundant than ABCB1, their expressions remain unchanged after drugs treatments (figure 6D and table S1).

## Discussion

Reference chemotherapies used in clinics demonstrated their impact on acquired resistance of tumor cells. The resistance phenotype can then be exploited by the tumor cells as a selective advantage to reconstitute the tumor mass [20]. In the present work focused on melanoma, we show that ABCB5^+^ cells have a survival advantage over the bulk of tumor cells when they are exposed to different cytotoxic compounds, including drugs that are used in melanoma treatment.

ABCB5 expression was initially detected in a subpopulation of a melanoma cell line (G3361), as well as in melanoma samples obtained from patients [10], [18]. Here, we re-examined ABCB5 cell-surface expression by flow cytometry in five melanoma cell lines using a different anti-ABCB5 antibody [21]. As previously shown elsewhere [14], [18], we noticed that a subpopulation of cells was consistently surface-labelled. We confirmed the specificity of this labelling by ABCB5 silencing experiments and co-labelling experiments with a different anti-ABCB5 antibody. The proportions of this ABCB5^+^ cells subset vary in the different cell lines but stay in a range that is similar to the frequency reported for the G3361 cell line [14]. This variability is in agreement with previous analyses of a panel of melanoma cell lines [13], [14], [21] and was confirmed when measuring the ABCB5 mRNA content by Q-PCR.

When examining the ABCB5 mRNA expressing cell lines, we found a significantly higher proportion of ABCB5^+^ cells in the WM-266-4 cell line that originates from a metastasis, compared to the WM-115 cells from the primary tumor of the same patient. Interestingly, it was previously shown that ABCB5 expression level is correlated with the grade of melanoma [18]. Our results suggest that the couple WM-115/WM-266-4 tumor cell lines might be an interesting *in vitro* model to study the role of the ABCB5 protein and of ABCB5-expressing cells in melanoma aggressiveness.

The numbers of ABCB5-expressing cells detected by flow cytometry correlate with the ABCB5 mRNA level except in the G-361 cell line. In WM-266-4 cells, we showed that inhibitors such as brefeldin A and cycloheximide affect this number. This suggests that, in addition to RNA level, expression of ABCB5 at the cell surface depends also on the rate of the protein synthesis and trafficking toward the cytoplasmic membrane. We thus can not exclude that some of these events are particularly enhanced in the G-361 cell line, leading to the detection of a significant number of ABCB5-expressing cells despite a low abundance of ABCB5 mRNA as measured on the total cell population.

As a member of the ABC transporters family, ABCB5 was suggested to participate to the chemoresistant phenotype of melanoma cells [1], [12], [14]. A direct role of ABCB5 as a functional ABC transporter was assayed with doxorubicin, using the fluorescent properties of this well known ABCB1 substrate [14], [16], [21]. Nevertheless the behaviour of the ABCB5^+^ subpopulation upon anti-melanoma drugs remains unknown. We addressed this question taking advantage of the anti-melanoma effect of temozolomide on subcutaneaous WM-266-4 tumors grafted in mice. Temozolomide is a pro-drug that, once metabolized, acts with a similar molecular mechanism and efficiency than dacarbazine [22]. Its therapeutic potency is well mimicked in our xenograft model since an objective tumor regression was measured. Here we show that, *in vivo*, the regression of melanoma tumor upon temozolomide treatment is associated with a selective survival and an increased number of cells that express ABCB5 protein on their surface.

In order to extend this observation to human patient tumors, we analyzed the ABCB5 expression in clinical samples. Since ABCB5 expression has been shown to vary according to the melanoma stage [18], we restricted our study to skin metastases from untreated patients and from patients who relapsed after a first chemotherapy episode. Interestingly, the highest levels of ABCB5 expression were found in metastases from treated patients. This result is consistent with our *in vivo* findings and provides additional support to the observation that ABCB5-expressing cells are enriched upon anti-melanoma treatment.

We investigated this apparent ABCB5^+^ cells enrichment *in vitro* by monitoring the absolute number of ABCB5-expressing cells. We focused our analysis on the effects of dacarbazine that was, until now, the reference treatment for metastatic melanoma [4] and vemurafenib that recently proved efficiency in melanoma with BRAF V600E mutation [6]. Using different cell lines that bear the BRAF V600E mutation, we showed that ABCB5^+^ cells survive to both drugs at doses that are efficient in killing ABCB5^−^ cells. The loss of viability detected at higher doses suggests that the mechanisms triggered to protect the cells from the cytotoxic stress are overwhelmed and, consequently, become inefficient. By contrast, ABCB5^+^ cells are more sensitive to the doxorubicin cytotoxic effect. *In vitro* cell treatments with dacarbazine were performed as long as two weeks (not shown). During the first week of treatment, we observed that the proportion of ABCB5-expressing cells continues to increase while the total number of cells decreases. After two weeks of treatment without interruption, proliferation resumes. Simultaneously, the proportion of ABCB5^+^ cells progressively decreases to a percentage that is similar to the percentage found in untreated cells. These results suggest the long-term surviving of cells and the recurrence of a heterogeneous population after a chemotherapeutic episode.

Both dacarbazine and doxorubicin stimulate the up-regulation of ABCB5 at the cell surface through protein neo-synthesis and trafficking. This might explain the slight difference consistently found between ABCB5^+^ cells and bulk cells cytotoxicity curves obtained with doxorubicin. But, as previously reported [23], we did not detect a doxorubicin-induced up-regulation by Western blot analysis since the frequency of ABCB5^+^ cells remains unchanged and stays very low after the treatment. Interestingly, dacarbazine has an additional effect leading to the neo-expression of ABCB5 at the surface of some cells. As previously reported with other substances [11], it up-regulates the ABCB5 mRNA level, but interacts also with the protein neo-synthesis. In addition, it induces the surface expression of ABCB5 on some cells through a process related to the intracellular vesicular traffic. The mechanisms underlying this observation remain to be understood.

We extended our analysis to a panel of cytotoxic compounds and found that drugs from various therapeutic classes induce a substantial increase in ABCB5^+^ cells in the surviving cellular population. One of the most potent chemoinducer is camptothecin. Interestingly, ABCB5 gene expression was found inversely correlated to camptothecin activity among cell lines of the NCI panel [14]. Furthermore, depletion using a siRNA that targets a short variant of ABCB5 was shown to sensitize melanoma cells to the camptothecin cytotoxic effect [12].

The peculiar cytotoxicity of doxorubicin on ABCB5-expressing cells remains to be understood. It suggests that the efficiency of ABCB5 as a transporter might be higher for drugs such as dacarbazine, vemurafenib, camptothecin, gemcitabine than for doxorubicin. To date, no transfection data are available to establish which substrates are preferentially transported by ABCB5. In addition, the length of the protein detected so far (isoform 2) raises unsolved questions about the three-dimensional structure of ABCB5 and its functionality as a transporter.

The implication of other ABC transporters in the melanoma resistance to chemotherapeutic drugs was extensively studied [24]–[28] and remains controversial. We wondered if various transporters might be associated with the selective survival of ABCB5^+^ cells. This hypothesis was assayed assuming that, if other ABC transporters were co-expressed by ABCB5^+^ cells, we should expect their concomitant enrichment with ABCB5 after drug treatment. Our data show here that the survival of ABCB5^+^ cells upon dacarbazine is not associated with ABCB1, ABCC1 or ABCG2 expression. An implication of ABCB1 in the response to drugs known as ABCB1 substrates can not be completely excluded, but does not appear predominant in the survival of ABCB5^+^ cells. A recent report suggests that a new member of the ABC family, ABCB8, is associated with doxorubicin resistance [29]. A similar expression level of such a transporter in ABCB5^+^ and ABCB5^−^ cells might explain the lack of differential sensitivity of these cells to doxorubicin but this was not investigated in our study.

Different studies suggest that, in addition to a plasma membrane insertion, some ABC transporters may also be located on subcellular compartments where they play a role in trapping drugs in the cytoplasm before export [30], [31]. The preferential ABCB5 expression in melanocytes and melanomas [11] pointed to a potential link between ABCB5 and the melanosomal compartment in which melanin is synthetized and stored. A role of melanosomes was proposed for melanoma cells chemoresistance to cisplatin [8], [32]. Nevertheless, the WM-266-4 cells lack a functional melanosomal compartment [33], suggesting that the survival of ABCB5^+^ during dacarbazine treatment is not associated with melanosomes. Moreover, ABCB5^+^ cells were also found in amelanic tissues [15], [16]. Thus the potential link of ABCB5 with this cellular detoxification pathway remains unclear.

Taken together, our results suggest that anti-melanoma chemotherapy participates to the chemoresistance acquisition that leads to clinical relapse, by selecting tumor cell subpopulations such as ABCB5-expressing cells. This is of particular importance in the chemoresistance occuring upon dacarbazine treatment, but is reinforced in the context of the clinical use of vemurafenib whose strong therapeutic interest is strongly hampered by rapid relapses observed after a few months [7].

ABCB5 appears as a dual potential therapeutic target [34]. On the one hand, the functions of the protein *per se* may confer tumor cells a survival advantage through the tumor progression and potentially the chemotherapeutic treatment. This last point was recently addressed in colorectal cancer in which ABCB5 was shown to identify a therapy-refractory tumor cell population [16]. On the other hand, the protein ABCB5 might not mechanistically participate to this selective survival, but represents a marker for a subcellular population endowed with intrinsic stem cell-like properties [18]. While the stemness of these cells remains controversial [35], their peculiar properties in terms of tumorigenicity [18], resistance and more recently, immunosuppression [36] remain to be further explored and monitoring the ABCB5^+^ cells might be an important parameter to evaluate the long term efficiency of melanoma treatment.

## Materials and Methods

### Cell Culture

The WM-266-4, WM-115, G-361, A-375 and SK-MEL-28 cell lines were obtained from the American Type Culture Collection and the KB and KB-V1 cell lines from the Deutsche Sammlung von Mikrooganismen und Zellkulturen. The WM-266-4, WM-115, G-361, A-375 and KB cells were grown in DMEM (Invitrogen, Cergy Pontoise, France) supplemented with 10% fetal bovine serum (Sigma, Lyon, France), 2 mM glutamine, 100 UI/mL penicillin-streptomycin and 1.25 µg/mL fungizone (both from Invitrogen) in 5% CO_2_ atmosphere. The SK-MEL-28 cells were grown in MEM (Invitrogen) supplemented with 10% fetal bovine serum, 2 mM glutamine, 100 U/mL penicillin-streptomycin, 1.25 µg/mL fungizone, 1 mM sodium pyruvate and 0.1 mM non essential amino acids (all from Invitrogen) in 5% CO_2_ atmosphere. The KB-V1 cells were grown in MEM (Invitrogen) supplemented with 15% fetal bovine serum, 2 mM glutamine, 100 U/mL penicillin-streptomycin, 1.25 µg/mL fungizone, 1 mM sodium pyruvate and 0.1 mM non essential amino acids (all from Invitrogen) and 200 ng/mL vinblastine in 5% CO_2_ atmosphere. Numerations of viable cells were performed using an Automated Cell Viability Analyzer (Beckman Coulter Vi-Cell).

### Flow Cytometry Analysis

The surface expression of ABCB5 and ABCB1 was analyzed respectively with a rabbit polyclonal anti-ABCB5 antibody ABCB5-Ab^Rock^ (Rockland, Gilbertsville, PA, USA) or a mouse monoclonal antibody ABCB5-Ab^AbD^ (clone 3C2-1D12, AbD Serotech, Oxford, UK) and a monoclonal anti-ABCB1 antibody (clone MM4.17, Millipore, Molsheim, France). Cells were detached with 2 mM EDTA in PBS and incubated for 45 min at 4°C with 6 µg/mL of ABCB5-Ab^Rock^ antibody, 10 µg/mL of ABCB5-Ab^AbD^ antibody, 5 µg/mL anti-ABCB1 or corresponding isotype antibodies (Invitrogen). The cells were washed, counter-stained with Alexa-488-conjugated goat anti-rabbit or Alexa-647-conjugated goat anti-mouse Ig antibodies (Invitrogen) and finally incubated with 0.5 µg/mL DAPI (Sigma). The cells were analyzed with a LSRII flow cytometer using the Diva software (both from BD Biosciences, Le Pont-De-Claix, France). ABCB5 and ABCB1 expression were monitored on live cells (gated as DAPI-negative cells).

### Si RNA-mediated Down-regulation of ABCB5 Expression

ABCB5 expression was silenced by transfection of siRNA with lipofectamine 2000 Reagent (Invitrogen) after 24 h of serum deprivation. ABCB5 siRNA and the control luciferase siRNA were from Applied Biosystem. The targeted ABCB5 sequence was 5′-ggucggacuacaaucgugg-3′. Cells were analyzed for ABCB5 expression 72 h after the transfection.

### Quantitative Real-Time PCR

RNA was prepared using the RNeasy Mini Kit (Qiagen, Courtaboeuf, France) and quantified by the NanoDrop technology (ThermoScientific). Total RNA (1 µg) was reverse transcribed into cDNA with the iScript cDNA Synthesis Kit (Bio-Rad). PCR were performed on an iCycler (Bio-Rad, Marne-la-Coquette, France) using the iQ SYBR Green Supermix (Bio-Rad) kit. All PCR included amplification of HMBS (hydroxymethylbilane synthase) and RPLP0 (ribosomal protein, large, P0) as normalizing controls. The primer sequences were designed as follows: ABCB5 Forward 5′-ccaaatcgggggctgcgcatctgtt-3′ and Reverse 5′-agccgctgctccccacaaatgcta-3′ for ABCB5 isoform 1 and 2; ABCB1 Forward 5′- tgacatttattcaaagttaaaagca-3′ and Reverse 5′- tagacactttatgcaaacatttcaa-3′; ABCC1 Forward 5′- agtggaacccctctctgtttaag-3′ and Reverse 5′- aacagcagcacggtgtagaa-3′; ABCG2 Forward 5′- ccgcgacagtttccaatgacct-3′ and Reverse 5′- gccgaagagctgctgagaactgta-3′; HMBS Forward 5′-atacagacggacagtgtggtgg-3′ and Reverse 5′-ccctgtggtggacatagcaatga-3′; RPLPO Forward 5′-ggcgacctggaagtccaact-3′ and Reverse 5′-ccatcagcaccacagccttc-3′. For each gene, the relative expression ratio was calculated by the ΔΔCt method.

### In *vivo Study*

Animals were handled and cared for in accordance with the Guide for the Care and Use of Laboratory Animals (National Research Council, 1996) and the European Directive EEC/86/609, under the supervision of authorized investigators. All experiments performed were controlled and approved by the institutional Animal Ethical Committee and by the “Comité Régional d’Ethique pour l’Expérimentation Animale” (Midi Pyrénées, France) (protocol approval number MP/05/16/04/06).

WM-266-4 cells (5×10^6^) were implanted subcutaneously into homozygous female athymic nude mice (Ico:Swiss-nu/nu, Charles River, Saint-Germain-sur-L’Arbresle, France) and allowed to increase to a median value of 100–200 mm^3^. After randomisation in treatment cages, temozolomide (Molekula, Dorset, UK) was administered i.p. as 4 intermittent injections over 8 days according to a [q2d×3] schedule at 80 mg/kg. In each chemotherapy trial, mice were checked daily, with any adverse clinical reactions noted and deaths recorded. Mice were weighed three times a week during the treatments. Tumors were measured by callipers three times weekly and tumor volumes (mm^3^) were estimated as = 0.5 (length×width×thickness). Results are presented for experiments involving 3–6 mice per experimental group. T/T0 (%) = mean of (tumor volume on day X of treatment/tumor volume on day 0 of treatment)×100.

The tumors were harvested and dissociated with collagenase (1000 UI/mL, Sigma), elastase (30 UI/mL, Sigma) and DNAse (1 mg/mL, Sigma) in culture medium. After filtration on 70 µm sieves, the cellular suspensions were labelled for cell surface ABCB5 then CD44 using an anti-human CD44 antibody conjugated to Alexa-647 (clone C26, Beckton Dickinson Pharmingen, San Diego, CA, USA) in order to distinguish tumor cells from the infiltrating host cells. ABCB5 labelling was analyzed on live cells (gated as DAPI-negative cells) expressing CD44. In addition, tumor samples from day 17 were paraffin-embedded for immunohistochemistry analyses.

### Clinical Cancer Specimens

A total of 15, formalin-fixed paraffin-embedded (FFPE) melanoma tumor samples from 8 patients were obtained after informed consent from the tumor bank of the Department of Pathology, Toulouse-Purpan University Hospital (France). Pathological specimens were collected between 2009 and 2011 and consisted of skin metastases. Tumor samples were collected before (n = 7) and after (n = 8) treatment of the melanoma with monochemotherapy (dacarbazine or fotemustine). All patients presented melanoma metastases at the time the tumor sample was taken (AJCC stage IV). In 3 patients, molecular analysis of the tumor revealed the presence of a BRAF V600E mutation, the mutation was absent in 2 patients. Due to technical artifact, the mutation was not evaluated in 3 patients. In 3 patients, a stable disease was observed after 1 cycle of monochemotherapy (dacarbazine).

### Immunohistochemistry

For immunohistochemistry, 4-µm sections were deparaffinized and rehydrated. Endogenous peroxide activity was inhibited with a 15 min incubation in 3% (v/v) hydrogen peroxide. An automated staining module (Dako, Glostrup, Denmark) was then used to conduct histological staining. Slides were incubated with a rabbit polyclonal anti-ABCB5 antibody ABCB5-Ab^Sigma^ (Sigma) followed by a secondary Horse Radish Peroxidase-rabbit antibody and visualized using a colorimetric method (Envision kit, Dako, Glostrup, Denmark). All of the colorimetric immunohistostained sections were analyzed using a Nikon (Eclipse E800) microscope. Semiquantitative double*-*blind evaluation was conducted by scoring the estimated staining intensity of the ABCB5-labelling.

### Cell Treatments with Chemotherapeutic Drugs

The WM-266-4 cells were seeded at 10^5^ cells per well in 24-well culture plates and treated after a 24 h period to allow cell attachment. The cells were harvested for analysis 72 h after the treatment onset and were counted using an Automated Cell Viability Analyzer (Beckman Coulter Vi-Cell) before flow cytometry experiments.

The half maximal effective concentration (EC50) of each drug was determined using a cytotoxicity assay (ATP-lite assay, Perkin Elmer, Courtaboeuf, France). The EC50 on WM-266-4 cells are: 3×10^−8^ M for vinblastine (Sigma), 3×10^−9^ M for vindesine (Lilly, Suresnes, France), 3×10^−9^ M for vincristine (Sigma), 3×10^−8^ M for paclitaxel (Sigma), 1×10^−5^ M for dacarbazine (Sigma), 5×10^−8^ M for doxorubicin (Sigma), 1×10^−6^ M for etoposide (Sicor, Irvin, CA, USA), 2×10^−8^ M for gemcitabine (Lilly), 2×10^−8^ M for camptothecin (Acros, Thermo-Fisher, Illkirch, France) and 4×10^−7^ M for vemurafenib (Euromedex, Selleckchem).

When mentioned, cycloheximide (10 µg/mL, Sigma) or brefeldin A (10 µg/mL, Sigma) were added respectively 24 h and 4 h before the end-points of the cytotoxic treatments.

### Western Blot Analysis

Cells were lysed with a Dounce homogenizer at a concentration of 10^7^ cells/mL in the lysis buffer (20 mM Tris-HCl, 150 mM NaCl, 5 mM MgCl2, 5 mM EGTA, 5 mM NaF, 1 mM NaVO4, 0.1% Nonidet-P40 and 0.1% Triton X114 complemented ex-temporaneously with 1 mM DTT and protease inhibitors cocktail (Calbiochem, Merck Chemical, Nottingham, UK). The unbroken cells and nuclei were pelleted by centrifugation at 600 g for 1 min and washed once with 100 µL of lysis buffer. The post-nuclear supernatant was centrifuged at 72000 g for 30 min. The membrane pellet was solubilised in 2% SDS, 15% glycerol, 50 mM dithiothreitol in 10 mM Tris pH 8, heated at 37°C for 1 h and sonicated. Samples were run on 8% SDS-PAGE and transferred onto polyvinylidene difluoruride membranes. After saturation with 5% dry milk in TBS-0.1%Tween 20, membranes were incubated with either anti-ABCB5 antibody (1/2000 diluted in TBS-0.1%Tween 20-1% BSA) or anti-actin antibody (clone C4, Millipore, 1/1000 in 5% dry milk). After wash, the membranes were revealed with peroxydase-linked secondary goat anti-mouse or rabbit antibody (Jackson ImmunoResearch, Suffolk, UK) and the signals were detected by ECL (GE Healthcare). Band quantification was performed with the Quantity One 1-D Analysis Software (Bio-Rad Laboratories, Inc.) and normalized using actin band intensities.

### Statistical Analysis

Data reported are expressed as means ± s.d. of at least three independent experiments. Statistical significance was measured using the Mann and Whitney sum rank test from SigmaStat3.1 software, where p<0.05 was considered to be statistically significant (marked with *) and p<0.01 and p<0.001 was considered highly statistically significant (marked respectively with ** and ***).

## Supporting Information

Figure S1 **Enrichment in ABCB5-expressing cells is associated with protein neo-synthesis and ABCB5 relocation at the cell-surface.** WM-266-4 cells were treated with doxorubicin or dacarbazine for 72 h. Cells were either untreated (**A**) or treated with brefeldin A (**B**) 4 h before the treatment end-point. A similar experiment was performed except that cells were either untreated (**C**) or treated with cycloheximide (**D**) 24 h before the treatment end-point. Cells were labelled for ABCB5 and analyzed by flow cytometry. Histograms show the fluorescence intensity of the ABCB5 cells in representative experiments. The corresponding mean of fluorescence intensity is indicated in each histogram.
(TIF)

Table S1 **Quantification of ABCB5, ABCB1, ABCC1 and ABCG2 mRNA expression after cytotoxic treatments.** WM-266-4 cells were treated with various drugs at their EC50 for 72 h. The relative mRNA expression of ABCB5, ABCB1, ABCC1, ABCG2 and HMBS as the house-keeping gene was measured by Q-PCR and the Ct values are reported.
(TIF)
