# Supplementary figures and images for: Melanoma Chemotherapy Leads to the Selection of ABCB5-Expressing Cells
Source: PLoS One. 2012 May 24;7(5):e36762. doi: 10.1371/journal.pone.0036762 (PMC3360047; doi:10.1371/journal.pone.0036762)

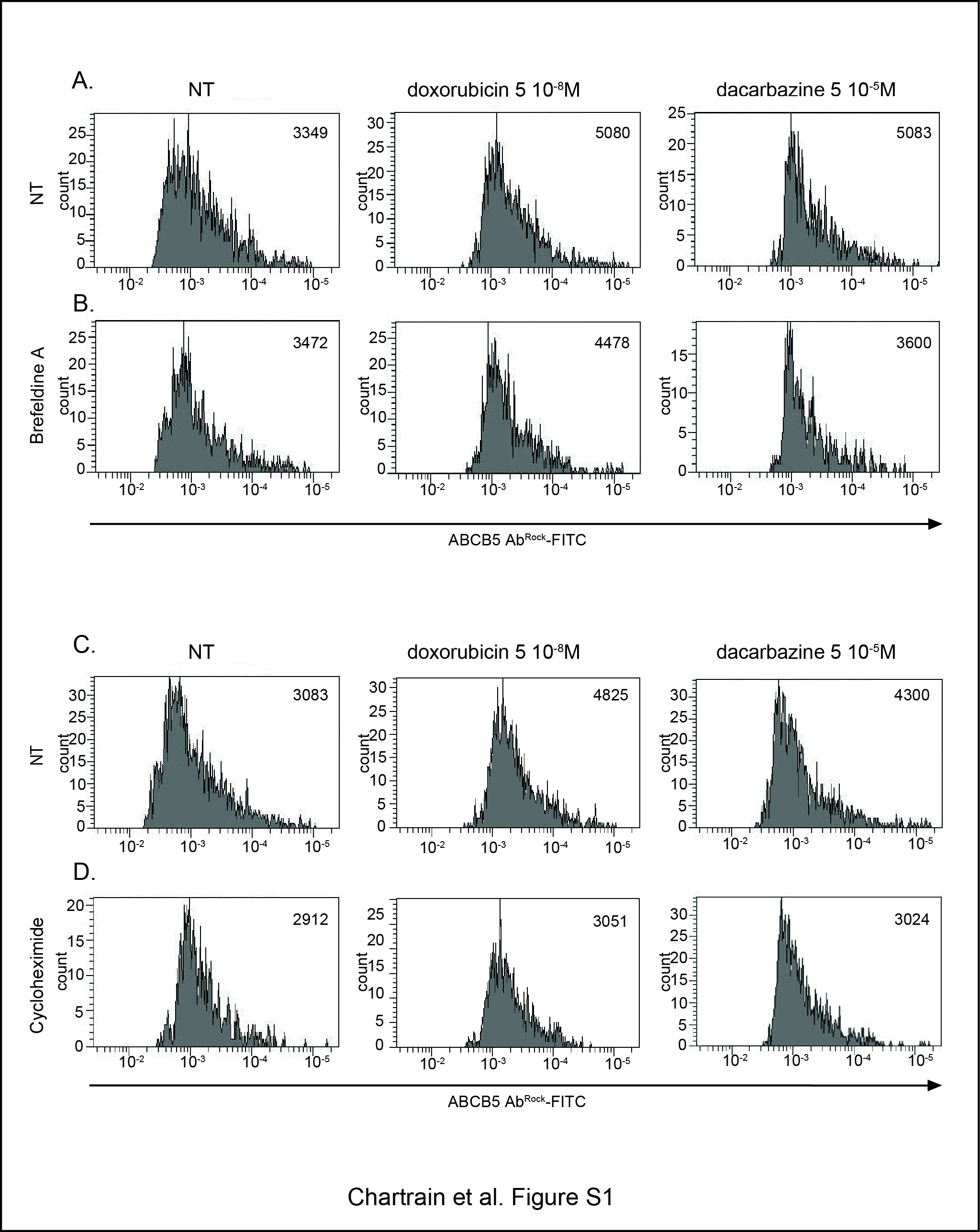

Supplement: Figure S1 — Enrichment in ABCB5-expressing cells is associated with protein neo-synthesis and ABCB5 relocation at the cell-surface. WM-266-4 cells were treated with doxorubicin or dacarbazine for 72 h. Cells were either untreated (A) or treated with brefeldin A (B) 4 h before the treatment end-point. A similar experiment was performed except that cells were either untreated (C) or treated with cycloheximide (D) 24 h before the treatment end-point. Cells were labelled for ABCB5 and analyzed by flow cytometry. Histograms show the fluorescence intensity of the ABCB5 cells in representative experiments. The corresponding mean of fluorescence intensity is indicated in each histogram. (TIF) [file pone.0036762.s001.tif]

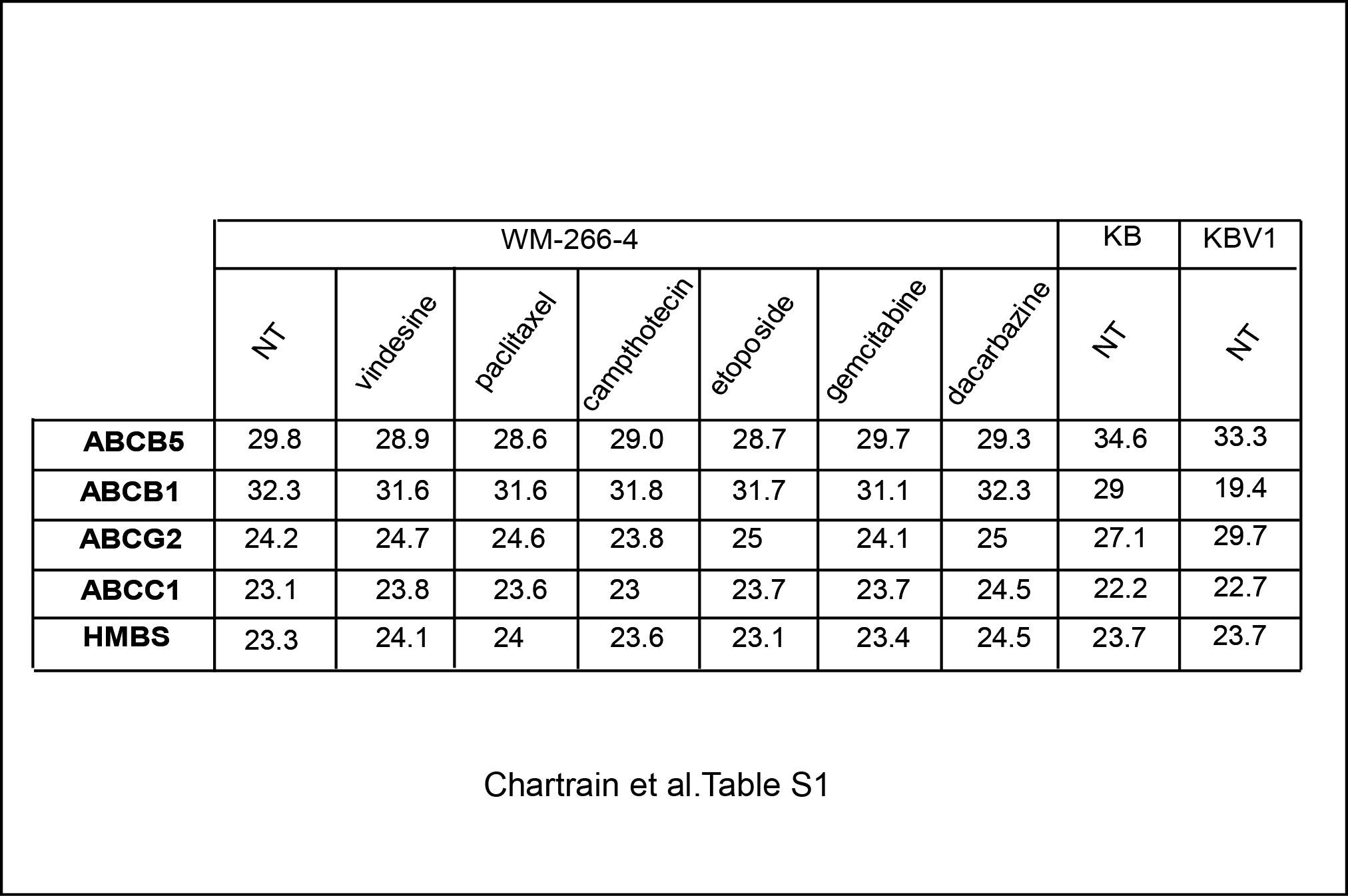

Supplement: Table S1 — Quantification of ABCB5, ABCB1, ABCC1 and ABCG2 mRNA expression after cytotoxic treatments. WM-266-4 cells were treated with various drugs at their EC50 for 72 h. The relative mRNA expression of ABCB5, ABCB1, ABCC1, ABCG2 and HMBS as the house-keeping gene was measured by Q-PCR and the Ct values are reported. (TIF) [file pone.0036762.s002.tif]
